# Custo-Efetividade das Terapias para Insuficiência Cardíaca no Brasil: Uma Revisão Sistemática

**DOI:** 10.36660/abc.20250712

**Published:** 2026-04-01

**Authors:** Guilherme Augusto Reissig Pereira, Mariana de Castro Lopes, Bernardo Ludwig Dama, Carisi Anne Polanczyk, Eduardo Gehling Bertoldi

**Affiliations:** 1 Hospital de Clínicas de Porto Alegre Porto Alegre RS Brasil Hospital de Clínicas de Porto Alegre, Porto Alegre, RS – Brasil; 2 Programa de Pós-Graduação em Cardiologia Universidade Federal do Rio Grande do Sul Porto Alegre RS Brasil Programa de Pós-Graduação em Cardiologia, Universidade Federal do Rio Grande do Sul (UFRGS), Porto Alegre, RS – Brasil; 3 Instituto Nacional de Ciência e Instituto de Avaliação de Tecnologia em Saúde Porto Alegre RS Brasil Instituto Nacional de Ciência e Instituto de Avaliação de Tecnologia em Saúde (IATS), Porto Alegre, RS – Brasil; 4 Hospital Moinhos de Vento Porto Alegre RS Brasil Hospital Moinhos de Vento, Porto Alegre, RS – Brasil; 5 Faculdade de Medicina Universidade Federal de Pelotas Pelotas RS Brasil Faculdade de Medicina, Universidade Federal de Pelotas (UFPEL), Pelotas, RS – Brasil

**Keywords:** Análise de Custo-Efetividade, Análise Custo-Benefício, Insuficiência Cardíaca

## Abstract

A insuficiência cardíaca (IC) figura entre as principais causas de mortalidade cardiovascular no Brasil. Em 2021, os custos atribuídos à IC ultrapassaram R$ 22 bilhões no Sistema Único de Saúde (SUS). A incorporação de novas tecnologias terapêuticas intensifica a necessidade de análises de custo-efetividade. Realizar uma revisão sistemática de análises econômicas relacionadas a terapias farmacológicas e não farmacológicas para IC no Brasil. A busca foi conduzida nas bases de dados PubMed, Embase, LILACS e SciELO. Foram incluídos estudos brasileiros que avaliaram custos e custo-efetividade de terapias na IC. Os desfechos analisados foram custo da doença, custo por anos de vida ajustados pela qualidade (QALY, na sigla em inglês) e custo por desfecho clínico. Foram incluídos 25 estudos. As análises de custo-efetividade utilizaram limiares de disposição a pagar derivados do produto interno bruto per capita, conforme recomendações da Comissão Nacional de Incorporação de Tecnologias no Sistema Único de Saúde. Sob a perspectiva do SUS, em pacientes com IC com fração de ejeção reduzida, espironolactona e eplerenona mostraram-se custo-efetivas, com razão de custo-efetividade incremental (RCEI) em dólares internacionais (Int$) de 7.955/QALY e Int$ 6.459/QALY, respectivamente. Dapagliflozina e sacubitril-valsartana apresentaram RCEI de Int$ 9.000/QALY e Int$ 11.691/QALY, respectivamente, sendo também consideradas custo-efetivas. A terapia de ressincronização cardíaca foi classificada como altamente custo-efetiva (RCEI de Int$ 15.723/QALY), enquanto o cardiodesfibrilador implantável para prevenção primária não demonstrou custo-efetividade (RCEI de Int$ 50.345/QALY). Diversas intervenções na IC apresentam atratividade econômica. Entretanto, a maioria das tecnologias foi avaliada em apenas um estudo, o que limita análises mais robustas. A ampliação de estudos econômicos nacionais é necessária para favorecer o acesso a terapias eficazes, mantendo a sustentabilidade do sistema de saúde.

## Graphical abstract

**Figura Central:**
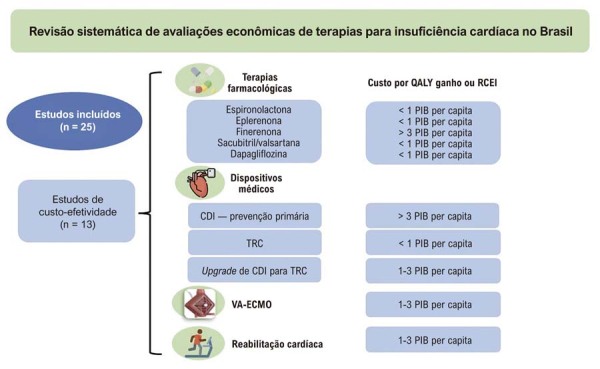
Custo-Efetividade das Terapias para Insuficiência Cardíaca no Brasil: Uma Revisão Sistemática

CDI: cardiodesfibrilador implantável; PIB: produto interno bruto; QALY: anos de vida ajustados pela qualidade (sigla em inglês); RCEI: razão de custo-efetividade incremental; TRC: terapia de ressincronização cardíaca; VA-ECMO: oxigenação por membrana extracorpórea venoarterial (sigla em inglês).

## Introdução

A insuficiência cardíaca (IC) constitui uma das principais causas de internação e mortalidade cardiovascular no Brasil. Em 2021, os custos diretos atribuídos à IC ultrapassaram R$ 22 bilhões no Sistema Único de Saúde (SUS), o que reflete não apenas a elevada prevalência da condição, mas também a crescente complexidade de seu manejo clínico. Entre 2008 e 2021, foram registradas mais de 3,4 milhões de hospitalizações por IC no SUS, o que corresponde a mais de um terço das admissões clínicas por causas cardiovasculares. Paralelamente, observou-se aumento na realização de procedimentos de alta complexidade, como revascularizações, trocas valvares, ablações de arritmias e implantes de dispositivos, o que evidencia a progressão da gravidade dos casos e o incremento dos custos associados às tecnologias utilizadas no manejo da IC. Os custos indiretos, incluindo perdas de produtividade e benefícios previdenciários, também são substanciais, estimados em aproximadamente R$ 6 bilhões por ano.^1 , 2^

Apesar da disponibilidade de novas terapias, o acesso e a adesão ao tratamento permanecem limitados, em grande parte devido ao custo dos medicamentos de uso crônico e contínuo, bem como ao custo dos procedimentos.^2^ De acordo com as normativas do sistema de saúde brasileiro, as recomendações para incorporação de tecnologias terapêuticas devem considerar não apenas eficácia, segurança e efetividade, mas também a custo-efetividade, de modo a subsidiar decisões informadas e eficientes. Embora existam estudos econômicos, ainda não há síntese estruturada que consolide as evidências sobre a custo-efetividade das terapias aplicadas à IC no contexto do sistema de saúde brasileiro.

Diante da magnitude do problema e da necessidade de alocação racional de recursos, este estudo foi conduzido para apoiar o processo de recomendação da Diretriz Brasileira de Insuficiência Cardíaca Crônica e Aguda, conduzida pela Sociedade Brasileira de Cardiologia. Para tanto, foi realizada uma revisão sistemática de análises econômicas sobre terapias para IC no Brasil.

## Métodos

### Estratégia de busca

Foi conduzida uma busca de alta sensibilidade nas bases PubMed, Embase, LILACS e SciELO para identificar estudos brasileiros que avaliaram custos e custo-efetividade de terapias farmacológicas e não farmacológicas no manejo da IC, publicados até 18 de março de 2025. As intervenções não farmacológicas incluíram procedimentos e dispositivos.

Foram utilizados descritores do *Medical Subject Headings* (ou equivalentes nas demais bases), como “economics”, “costs and cost analysis”, “value of life” e “heart failure”, além de termos livres, incluindo “cost”, “price”, “pharmacoeconomic”, “budget” e “money value”.

### Critérios de elegibilidade

Os estudos foram considerados elegíveis quando atenderam aos seguintes critérios: i) população composta por indivíduos com diagnóstico de IC, independentemente da fração de ejeção (FE), idade, sexo ou etiologia; ii) intervenção correspondente a qualquer terapia validada para o manejo da IC; iii) comparação com outro tratamento ativo, placebo ou ausência de intervenção; iv) desfechos relacionados ao custo da doença em dólares internacionais (Int$), custo por anos de vida ajustados pela qualidade (QALY, na sigla em inglês) e custo por desfechos clínicos (p.ex., hospitalização e mortalidade); v) delineamento incluindo análises econômicas de custo-efetividade, custo-utilidade ou custo da doença.

Revisões, relatos de caso, cartas ao editor, editoriais, estudos sem análise econômica explícita, estudos sem dados relevantes para a presente revisão e estudos realizados fora do cenário brasileiro foram excluídos da análise.

### Seleção dos estudos e extração dos dados

A seleção dos estudos foi realizada por meio da plataforma Covidence.^3^ Após a remoção de duplicatas, quatro revisores independentes realizaram a triagem de títulos e resumos. Os artigos elegíveis foram submetidos à leitura completa por dois revisores independentes. Divergências quanto à inclusão ou exclusão foram resolvidas por consenso ou mediante consulta a um terceiro revisor.

### Avaliação da qualidade metodológica

A qualidade metodológica dos estudos incluídos foi avaliada utilizando a lista *Consensus on Health Economic Criteria* (CHEC),^4^ composta por 19 itens que contemplam aspectos essenciais de avaliações econômicas baseadas em ensaios clínicos. Cada item foi classificado como “Sim”, “Não” ou “Não se aplica”, com uma justificativa registrada. A avaliação foi conduzida de forma independente por dois revisores, com resolução de divergências por consenso ou por um terceiro avaliador.

### Apresentação dos resultados

Diante da heterogeneidade metodológica observada entre os estudos incluídos, os resultados foram sintetizados de forma narrativa, contemplando a descrição da perspectiva analítica, métodos utilizados, fontes de dados e principais achados. Os estudos foram agrupados em terapias farmacológicas e não farmacológicas, e a qualidade da evidência dessas intervenções foi descrita em detalhe.

A análise dos resultados de razão de custo-efetividade incremental (RCEI) em relação ao limiar de disposição a pagar (LDP) foi conduzida de forma qualitativa. Os custos foram expressos em Int$, ajustados pela paridade do poder de compra (PPC) correspondente ao ano de realização de cada estudo, sem aplicação de correção inflacionária. Os valores foram interpretados em relação ao produto interno bruto (PIB) per capita e ao LDP vigente no ano de publicação.

Em 2024, o PIB per capita do Brasil ajustado pela PPC foi de 19.648 Int$. Foram utilizadas como fontes oficiais o Instituto Brasileiro de Geografia e Estatística para valores per capita em reais dos últimos 25 anos^5^ e dados de *trade marketing* para conversão em Int$.^6^

Quanto ao LDP, em consonância com o documento orientador da Comissão Nacional de Incorporação de Tecnologias no Sistema Único de Saúde (CONITEC),^7^ considerou-se custo-efetiva a terapia com valor inferior a 1 vez o PIB per capita. Valores entre 1 e 3 vezes o PIB per capita foram classificados como custo-efetivos em cenários especiais, como ausência de alternativa terapêutica, doenças graves, raras ou oncológicas. Valores superiores a 3 vezes o PIB per capita foram considerados indicativos de não custo-efetividade.

## Resultados

### Características dos estudos selecionados

**Figura 1:**
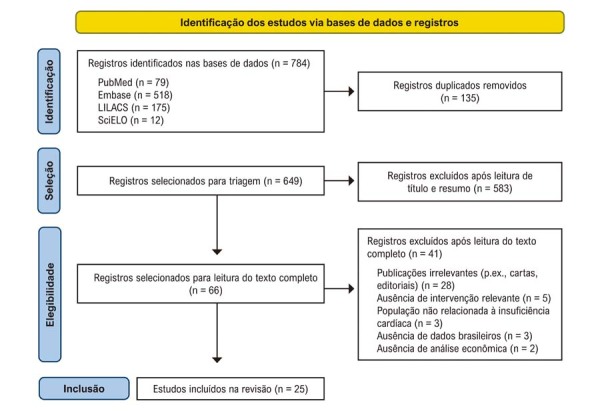
– Fluxograma de seleção de artigos para revisão.

A busca identificou 784 artigos. Após a remoção de duplicatas e a triagem por título e resumo, 66 publicações foram selecionadas para leitura completa. Destas, 25 foram incluídas na revisão sistemática ( Figura 1 ). Entre os estudos incluídos, 10 avaliaram custo da doença e 13 investigaram custo-efetividade ( Figura Central ), incluindo três documentos da CONITEC, além de uma revisão de literatura e uma revisão sistemática.

### Tratamento farmacológico da insuficiência cardíaca

Foram identificados diversos estudos que avaliaram a custo-efetividade de terapias consideradas essenciais no manejo da IC. A Tabela 1 descreve as características das análises econômicas de terapias farmacológicas conduzidas no Brasil sob a perspectiva do SUS. Em relação aos medicamentos que reduzem mortalidade na IC com FE reduzida (ICFEr), foram selecionados estudos envolvendo antagonistas do receptor mineralocorticoide (ARMs), inibidores do cotransportador sódio-glicose 2 (iSGLT2s) e inibidores de neprilisina-receptor de angiotensina (INRAs). Não foram identificados estudos no contexto de IC com FE preservada (ICFEp).

**Tabela 1:** – Análises econômicas de terapia farmacológica em pacientes com IC no Brasil

ID do estudo	Análise econômica	População	Intervenção	Comparação	Desfechos	Perspectiva
CONITEC, 2018^11^	Custo-efetividade	≥ 18 anos, IC NYHA II-IV e FEVE ≤ 35%	Sacubitril/valsartana	Tratamento padrão com IECAs ou BRAs	RCEI de Int$ 11.691/QALY	Sistema de saúde público
CONITEC, 2022^7^	Custo-efetividade	≥ 18 anos, IC NYHA II-IV com FEVE ≤ 40%, em tratamento padrão com IECAs/BRAs, betabloqueadores, diuréticos e ARMs	Dapagliflozina	Tratamento padrão (IECAs/BRAs, betabloqueadores, diuréticos e ARMs)	RCEI de Int$ 3.907/QALY	Sistema de saúde público
Koeche et al., 2025^8^	Custo-efetividade	≥ 18 anos com ICFEr, NYHA II-IV	Espironolactona	Tratamento padrão	RCEI de Int$ 7.955/QALY	Sistema de saúde público
			Eplerenona		RCEI de Int$ 6.459/QALY	
			Finerenona		RCEI de Int$ 109.840/QALY	
Naves et al., 2025^9^	Custo-efetividade	≥ 18 anos, IC NYHA II-IV com FEVE ≤ 40%, em tratamento padrão com IECAs/BRAs, betabloqueadores, diuréticos e ARMs	Dapagliflozina	Tratamento padrão	RCEI de Int$ 9.000/QALY	Sistema de saúde público

ARMs: antagonistas do receptor mineralocorticoide; BRAs: bloqueadores do receptor de angiotensina II; CONITEC: Comissão Nacional de Incorporação de Tecnologias no Sistema Único de Saúde; FEVE: fração de ejeção do ventrículo esquerdo; IC: insuficiência cardíaca; ICFEr: IC com fração de ejeção reduzida; IECAs: inibidores da enzima conversora de angiotensina; Int$: dólar internacional; NYHA: New York Heart Association; QALY: anos de vida ajustados pela qualidade (sigla em inglês); RCEI: razão de custo-efetividade incremental.

Espironolactona, eplerenona e finerenona foram avaliadas em pacientes com ICFEr de etiologia isquêmica e não isquêmica em um estudo de custo-efetividade.^8^ Entre os ARMs, espironolactona e eplerenona foram consideradas estratégias custo-efetivas, com custo por QALY ganho — ou RCEI — de Int$ 7.955/QALY e Int$ 6.459/QALY, respectivamente, valores inferiores ao LDP correspondente a 1 vez o PIB per capita. Nas análises de sensibilidade probabilísticas, espironolactona e eplerenona demonstraram robustez, com probabilidade de custo-efetividade em 87% e 92% das iterações, respectivamente. Em contraste, a finerenona foi considerada não custo-efetiva, com RCEI de Int$ 109.840/QALY, substancialmente acima do LDP adotado pelo Ministério da Saúde.

Entre os ISGLT2, a dapagliflozina mostrou-se estratégia economicamente favorável independentemente da presença de diabetes mellitus. A incorporação ao tratamento padrão da ICFEr resultou em ganho adicional de 0,36 QALY e RCEI de Int$ 9.000/QALY. A análise da curva de aceitabilidade indicou que, considerando LDP de Int$ 17.250, a probabilidade de custo-efetividade foi de 96,6%.^9^

A avaliação econômica dos INRAs baseou-se em subpopulação latino-americana do ensaio clínico randomizado (ECR) PARADIGM-HF,^10^ buscando maior representatividade para a população brasileira. Comparado ao enalapril, o uso de INRAs proporcionou ganho de 0,57 QALY e RCEI de Int$ 11.691/QALY, sendo classificado como estratégia custo-efetiva.^11^ Recomendações baseadas em valor em saúde para terapias farmacológicas são apresentadas na Tabela 2 .

**Tabela 2:** – Custo-efetividade e valor em saúde na terapia farmacológica da ICFEr

Terapia	Custo-efetividade qualitativa	Nível de certeza	Recomendação
Espironolactona e eplerenona	Dominante ou RCEI < Int$ 19.648*/QALY	Alto	Alto valor em saúde no tratamento de pacientes com ICFEr sintomática
Finerenona	RCEI > Int$ 58.944^†^/QALY	Baixo	Baixo valor em saúde no tratamento de pacientes com ICFEr sintomática
Dapagliflozina	Dominante ou RCEI < Int$ 19.648*/QALY	Alto	Alto valor em saúde no tratamento de pacientes com ICFEr sintomática
Sacubitril + valsartana	Dominante ou RCEI < Int$ 19.648*/QALY	Alto	Alto valor em saúde no tratamento de pacientes com ICFEr sintomática

*PIB per capita no Brasil em 2024 ajustado por PPC. ^†^Três vezes o PIB per capita no Brasil em 2024 ajustado por PPC. ICFEr: insuficiência cardíaca com fração de ejeção reduzida; Int$: dólar internacional; PIB: produto interno bruto; PPC: paridade do poder de compra; QALY: anos de vida ajustados pela qualidade (sigla em inglês); RCEI: razão de custo-efetividade incremental.

Até o momento, não foram identificadas análises econômicas nacionais de outras terapias farmacológicas redutoras de mortalidade na ICFEr, como inibidores da enzima conversora de angiotensina (IECAs), betabloqueadores e bloqueadores do receptor de angiotensina II (BRAs).

Além dos custos farmacológicos, o acompanhamento do paciente com IC envolve exames, honorários de profissionais de saúde, transporte e infraestrutura assistencial. Um estudo nacional publicado em 2024, que utilizou o método de custeio baseado em atividades orientado pelo tempo, demonstrou custos médios de Int$ 398 para pacientes em classe funcional *New York Heart Association* (NYHA) I, Int$ 548 para NYHA II e Int$ 1.233 para NYHA III/IV, com exames laboratoriais e de imagem representando até 74% dos gastos na classe III/IV.^12^

### Tratamento não-farmacológico da insuficiência cardíaca

Em relação às intervenções não farmacológicas ( Tabela 3 ), a reabilitação cardíaca, ou exercício supervisionado, é segura em pacientes com IC estável. No modelo de Kühr et al.,^13^ assumiu-se a realização de exercício três vezes por semana durante 3 meses, seguida de uma sessão semanal por 9 meses, o que totaliza 72 sessões no primeiro ano. Comparada ao cuidado usual, a reabilitação cardíaca apresentou RCEI de Int$ 26.462/QALY. Na análise de sensibilidade por simulação de Monte Carlo, considerando LDP de Int$ 27.500, a intervenção foi custo-efetiva em 55% das iterações.

**Tabela 3:** – Análises econômicas de terapia não farmacológica em pacientes com IC no Brasil

ID do estudo	Análise econômica	População	Intervenção	Comparação	Desfechos	Perspectiva
Kühr et al., 2011^13^	Custo-efetividade	≥ 60 anos, IC NYHA II-III	Exercício supervisionado	Tratamento padrão	RCEI de Int$ 26.462/QALY	Sistema de saúde público
Ruschel et al., 2018^15^	Custo-efetividade	≥ 18 anos, FE ≤ 45%, hospitalização por IC descompensada	Visitas domiciliares e chamadas telefônicas por enfermeiras	Tratamento padrão	RCEI de Int$ 265 por reinternação evitada	Sistema de saúde público
Ruschel et al., 2018^15^	Custo-efetividade	≥ 18 anos, FE ≤ 45%, hospitalização por IC descompensada	Visitas domiciliares e chamadas telefônicas por enfermeiras	Tratamento padrão	Dominante (menor custo e mais efetivo)	Sistema de saúde privado
Ghisleni et al., 2023^12^	Custo de doença	≥ 18 anos, IC com FE < 50%	Consultas e exames ambulatoriais	—	Custo médio em 6 meses: Int$ 398 (NYHA I); Int$ 548 (NYHA II); Int$ 1.233 (NYHA III/IV)	Sistema de saúde público
Goldraich et al., 2018^18^	Custo de doença	IC avançada, receptores de transplante cardíaco	Transplante cardíaco	—	Custo médio de Int$ 107.390 por paciente transplantado	Sistema de saúde público
Ribeiro et al., 2010^20^	Custo-efetividade	≥ 60 anos, FE < 35%, IC NYHA II-III	CDI (prevenção primária)	Tratamento padrão	RCEI de Int$ 50.345/QALY	Sistema de saúde público
Bertoldi et al., 2013^21^	Custo-efetividade	FE < 35%, IC NYHA II-IV, bloqueio de ramo	TRC	Tratamento padrão	RCEI de Int$ 15.723/QALY	Sistema de saúde público
Bertoldi et al., 2013^21^	Custo-efetividade	FE < 35%, IC NYHA II-IV, bloqueio de ramo	CDI e upgrade para TRC	CDI	RCEI de Int$ 36.940/QALY	Sistema de saúde público
Wherry et al., 2021^22^	Custo-efetividade	Pacientes de maior risco arrítmico*	CDI	Tratamento padrão	RCEI de Int$ 9.575/QALY	Sistema de saúde público
Decker et al., 2024^17^	Custo-efetividade	≥ 18 anos, choque cardiogênico refratário	VA-ECMO	Tratamento padrão	RCEI de Int$ 37.491/QALY; RCEI de Int$ 27.432 por ano de vida ganho	Sistema de saúde público

*Pacientes com pelo menos uma das seguintes variáveis: taquicardia ventricular não sustentada, ectopias ventriculares frequentes, FE < 25% ou pré-síncope/síncope. CDI: cardiodesfibrilador implantável; FE: fração de ejeção; IC: insuficiência cardíaca; Int$: dólar internacional; NYHA: New York Heart Association; QALY: anos de vida ajustados pela qualidade (sigla em inglês); RCEI: razão de custo-efetividade incremental; TRC: terapia de ressincronização cardíaca; VA-ECMO: oxigenação por membrana extracorpórea venoarterial (sigla em inglês).

A estratégia de visitas domiciliares regulares realizadas por enfermeiras treinadas, associadas a contato telefônico por 24 semanas, foi avaliada em um ECR no Brasil e demonstrou redução de 27% no desfecho composto de morte, atendimento em emergência ou reinternação.^14^ Sob a perspectiva do SUS, a intervenção apresentou RCEI de Int$ 265 por reinternação evitada; sob a perspectiva da saúde suplementar, mostrou-se dominante, com menor custo e menor taxa de reinternação.^15^

Em pacientes graves com risco iminente de morte, admite-se LDP de até 3 vezes o PIB per capita. No choque cardiogênico refratário, pode ocorrer escalonamento para dispositivos de suporte circulatório mecânico, incluindo a oxigenação por membrana extracorpórea venoarterial (VA-ECMO, na sigla em inglês). Um ECR recente não demonstrou benefício em mortalidade com VA-ECMO em choque cardiogênico pós-infarto quando comparado à terapia padrão,^16^ o que mantém o debate acerca de sua indicação. Em análise econômica conduzida no Brasil sob a perspectiva do SUS, o uso de VA-ECMO em pacientes com choque cardiogênico apresentou RCEI de Int$ 37.491/QALY em comparação à terapia padrão, considerando valores de LDP entre 1-3 vezes o PIB per capita. Esse resultado mostrou-se particularmente sensível ao custo de hospitalização e à probabilidade de sobrevivência dos pacientes submetidos ao VA-ECMO.^17^

Em pacientes com IC avançada, um estudo de custo realizado em 2018 com 27 receptores de transplante cardíaco em centro de referência identificou custo médio de Int$ 107.390 por paciente, considerando a internação hospitalar associada ao transplante. Esse valor excedeu em aproximadamente 60% o reembolso do SUS por paciente.^18^

O uso de dispositivos cardíacos tem aumentado no manejo da IC. Estudos sobre cardiodesfibrilador implantável (CDI), conduzidos sob a perspectiva do SUS e da saúde suplementar, indicaram baixa atratividade econômica na prevenção primária de arritmias ventriculares, independentemente da etiologia. Entretanto, o CDI mostrou-se custo-efetivo quando indicado para indivíduos com maior risco arrítmico, especialmente na IC isquêmica e em prevenção secundária.^19 , 20^

Quanto à terapia de ressincronização cardíaca (TRC), um estudo sob a perspectiva do SUS demonstrou que TRC isolada é altamente custo-efetiva em pacientes com IC, FE < 35%, classe funcional NYHA II-IV e dissincronia (bloqueio do ramo esquerdo). O *upgrade* para TRC associada ao CDI não foi custo-efetivo na população ampla, exigindo seleção criteriosa de pacientes com maior risco arrítmico.^21^

Análise econômica adicional avaliou o implante de CDI em subgrupo de pacientes com fatores de risco adicionais, incluindo taquicardia ventricular não sustentada, extrassístoles ventriculares frequentes, FE < 25% ou síncope/presíncope. Nesse grupo de maior risco, o CDI foi custo-efetivo, com custo/QALY inferior aos LDPs usuais.^22^

### Qualidade da evidência

A avaliação metodológica dos 25 estudos revelou variabilidade na qualidade. Seis estudos atenderam a 18 dos 19 critérios da lista CHEC, correspondendo a 94,7% de conformidade, enquanto três atingiram 89,4%, indicando boa aderência aos princípios das avaliações econômicas. A menor qualidade metodológica observada em parte dos estudos relaciona-se ao fato de que 12 não constituíam análises completas de custo-efetividade, o que limita a aplicabilidade de diversos itens da lista CHEC. Detalhes adicionais sobre a avaliação da qualidade metodológica são apresentados no Apêndice Suplementar.

## Discussão

A incorporação de novas terapias no SUS depende de análises econômicas conduzidas conforme os critérios estabelecidos pela CONITEC, constituindo componente obrigatório dos dossiês de avaliação de tecnologias. A perspectiva do SUS geralmente adota horizonte temporal de médio a longo prazo, considerando custos diretos do sistema e impactos sobre desfechos clínicos, como hospitalizações, mortalidade e qualidade de vida, mensurada por QALY ou anos de vida ajustados por incapacidade. Recentemente, a CONITEC passou a adotar limiares de custo-efetividade próximos a 1 vez o PIB per capita (R$ 40.000/QALY em 2022) como valor em saúde alto (desejável) e próximos a 3 vezes o PIB per capita (R$ 120.000/QALY em 2022) como valor baixo, com valores intermediários entre essas referências.^7^ Ambos os órgãos reguladores têm incorporado progressivamente princípios de cuidados em saúde baseada em valor, com o objetivo de promover maior eficiência, equidade e racionalidade na alocação de recursos.

O tratamento farmacológico da IC tem como objetivos centrais reduzir mortalidade, hospitalizações e sintomas. No contexto da ICFEr, terapias com impacto consistente nesses desfechos incluem IECAs, BRAs, INRAs, betabloqueadores, ARMs e iSGLT2s. Em razão do benefício clínico expressivo dessas intervenções, a maior parte das análises de custo-efetividade concentra-se na ICFEr. No Brasil, estudos com ARMs, iSGLT2s e INRAs demonstraram RCEI inferior a 1 vez o PIB per capita, o que indica que essas terapias apresentam alto valor em saúde no cenário nacional.

Por outro lado, na ICFEp, a menor disponibilidade histórica de terapias com impacto consistente em desfechos clínicos relevantes contribui para a ausência de avaliações econômicas específicas no país. Evidências recentes envolvendo iSGLT2s, finerenona e terapias voltadas à redução de peso sugerem que estudos econômicos nessa população devem emergir nos próximos anos.

Apesar da eficácia consolidada de betabloqueadores, IECAs e BRAs na redução de desfechos cardiovasculares, não foram identificadas análises econômicas dessas terapias no contexto brasileiro. Em outros países, avaliações conduzidas nas décadas de 1990 e 2000 demonstraram resultados favoráveis. Enalapril, captopril e ramipril mostraram-se opções economicamente viáveis na ICFEr; especificamente, o enalapril apresentou RCEI de US$ 115/QALY em comparação ao placebo em pacientes com FE do ventrículo esquerdo (FEVE) ≤ 35%.^23^ De forma semelhante, o uso de valsartana na disfunção sistólica pós-infarto demonstrou RCEI de £ 5.300/QALY, abaixo do LDP adotado no Reino Unido.^24^

Avaliações econômicas com betabloqueadores foram realizadas nos Estados Unidos, Itália e Reino Unido. Em uma dessas análises, o succinato de metoprolol apresentou RCEI de US$ 4.200/QALY em comparação ao placebo,^25^ demonstrando custo-efetividade, inclusive em indivíduos com idade superior a 70 anos.^26^

Outras terapias adjuvantes na ICFEr sintomática também foram avaliadas. No sistema de saúde americano, a combinação de hidralazina e dinitrato de isossorbida em pacientes negros com ICFEr sintomática mostrou-se estratégia dominante, isto é, mais eficaz e menos custosa que o tratamento convencional.^27^ No Reino Unido, a reposição de carboximaltose férrica IV em pacientes com ICFEr sintomática e deficiência de ferro, com ou sem anemia, apresentou RCEI de € 4.414/QALY, valor substancialmente inferior ao LDP adotado no país (€ 22.200-33.300/QALY).^28^

A ivabradina, indicada para pacientes com ICFEr classe funcional NYHA II-III em ritmo sinusal, frequência cardíaca ≥ 70 bpm e uso de dose máxima tolerada de betabloqueador,^29^ foi avaliada em estudo americano, com RCEI estimada de US$ 28.318/QALY, sendo classificada como intervenção de valor intermediário em saúde.^30^ O vericiguat, estimulador da guanilato ciclase solúvel, demonstrou redução do desfecho composto de morte cardiovascular e hospitalização por IC no estudo VICTORIA.^31^ Uma análise econômica subsequente estimou RCEI de US$ 124.512/QALY, sendo classificado como terapia de valor intermediário na população americana.^32^

Em relação à digoxina, estudo da década de 1990 nos Estados Unidos indicou redução de custos com sua manutenção em comparação ao tratamento padrão da época. Contudo, a interpretação desses resultados deve ser cautelosa, uma vez que terapias atualmente consideradas pilares do tratamento da ICFEr, como betabloqueadores e ARMs, ainda não eram amplamente utilizadas naquele período.^33^

No Brasil, os custos relacionados ao transplante cardíaco apresentam particular relevância. O custo médio de Int$ 107.390 por paciente é semelhante ao observado na Europa e aproximadamente metade do valor médio reportado nos Estados Unidos. À época da publicação, o reembolso do Ministério da Saúde era de Int$ 26.010 por procedimento, significativamente inferior ao custo real.^18^

A abordagem multidisciplinar é fundamental para o engajamento e a adesão de pacientes com IC. Estratégias como visitas domiciliares pós-alta por profissionais treinados e programas de reabilitação cardíaca têm sido amplamente recomendadas. Embora um ECR tenha demonstrado redução discreta de mortalidade e hospitalização apenas após ajuste por variáveis prognósticas,^34^ uma análise econômica brasileira indicou custo por QALY entre 1-3 vezes o PIB per capita, o que, em saúde, é um valor intermediário.

A avaliação econômica de dispositivos implantáveis apresenta desafios específicos. Esses dispositivos podem oferecer benefício clínico relevante em ampla gama de pacientes, porém sua custo-efetividade varia conforme o perfil clínico e o risco basal da população. Além disso, o padrão de custos não linear, com despesas iniciais elevadas relacionadas ao implante, dificulta sua adoção indiscriminada nos sistemas de saúde.

Os estudos econômicos sobre CDI e TRC no Brasil foram majoritariamente publicados na década de 2010. Avanços tecnológicos e mudanças no tratamento padrão desde então podem modificar a custo-efetividade dessas intervenções, tanto pelo impacto nos custos e na durabilidade dos dispositivos quanto pela alteração do risco basal dos pacientes.

Até onde se sabe, esta constitui a primeira revisão sistemática de análises econômicas sobre terapias para IC no Brasil. Observa-se crescente reconhecimento do papel das análises de custo-efetividade como complemento aos dados de eficácia clínica na incorporação de intervenções nos sistemas público e privado. Ainda assim, persistem lacunas relevantes na produção de evidências econômicas nacionais em IC.

### Limitações do estudo

Este estudo apresenta algumas limitações. Primeiramente, para a maioria das terapias existe apenas uma análise econômica disponível, o que reduz a robustez das conclusões e limita a generalização para diferentes perfis populacionais e contextos assistenciais. Essa escassez também restringe a aplicação de metodologias de avaliação da certeza da evidência (p.ex., *Grading of Recommendations Assessment, Development and Evaluation* ) que dependem de múltiplas fontes.

Em segundo lugar, diversos modelos utilizaram dados internacionais para estimar desfechos de eficácia, especialmente nas análises mais antigas. As adaptações necessárias nesse processo exigem cautela na extrapolação dos resultados, considerando diferenças entre sistemas de saúde, epidemiologia e adesão ao tratamento.

Por fim, a ausência de análises de custo-efetividade em ICFEp no Brasil impede recomendações específicas para esse subgrupo.

## Conclusão

Esta revisão sistemática demonstra que múltiplas intervenções com impacto clínico na IC apresentam atratividade econômica, com razões de custo por QALY favoráveis mesmo em cenários de restrição orçamentária. Identificou-se, contudo, importante lacuna na disponibilidade de análises econômicas conduzidas sob a perspectiva brasileira. A ampliação da produção de estudos econômicos nacionais pode favorecer o acesso equitativo às melhores intervenções disponíveis, promover o uso racional dos recursos e contribuir para a sustentabilidade dos sistemas de saúde, ao mesmo tempo em que melhora os desfechos clínicos dos pacientes.
