# Supplementary material for: Custo-Efetividade das Terapias para Insuficiência Cardíaca no Brasil: Uma Revisão Sistemática
Source: Arq Bras Cardiol. 2026 Apr 1;123(3):e20250712. [Article in Portuguese] doi: 10.36660/abc.20250712 (PMC13128211; doi:10.36660/abc.20250712)
Supplement: Translation

# Translation: Cost-Effectiveness of Therapies for Heart Failure in Brazil: A Systematic Review

**DOI:** 10.36660/abc.20250712i

**Authors:** Guilherme Augusto Reissig Pereira, Mariana de Castro Lopes, Bernardo Ludwig Dama, Carisi Anne Polanczyk, Eduardo Gehling Bertoldi

**Affiliations:** 1 Brazil Hospital de Clínicas de Porto Alegre, Porto Alegre, RS – Brazil; 2 Brazil Programa de Pós-Graduação em Cardiologia, Universidade Federal do Rio Grande do Sul (UFRGS), Porto Alegre, RS – Brazil; 3 Brazil Instituto Nacional de Ciência e Instituto de Avaliação de Tecnologia em Saúde (IATS), Porto Alegre, RS – Brazil; 4 Brazil Hospital Moinhos de Vento, Porto Alegre, RS – Brazil; 5 Brazil Faculdade de Medicina, Universidade Federal de Pelotas (UFPEL), Pelotas, RS – Brazil

**Keywords:** Cost-Effectiveness Analysis, Cost-Benefit Analysis, Heart Failure

## Abstract

Heart failure (HF) is among the leading causes of cardiovascular mortality in Brazil. In 2021, costs attributable to HF exceeded R$ 22 billion in the Brazilian Unified Health System (SUS). The incorporation of new therapeutic technologies increases the need for cost-effectiveness analyses. To perform a systematic review of economic analyses related to pharmacological and nonpharmacological therapies for HF in Brazil. Searches was conducted in the PubMed, Embase, LILACS, and SciELO databases. Brazilian studies that evaluated costs and cost-effectiveness of therapies in HF were included. The analyzed outcomes were disease cost, cost per quality-adjusted life years (QALY), and cost per clinical outcome. A total of 25 studies were included. Cost-effectiveness analyses used willingness-to-pay thresholds derived from gross domestic product per capita, according to recommendations of the National Committee for Health Technology Incorporation in the Brazilian Unified Health System. From the SUS perspective, in patients with HF with reduced ejection fraction, spironolactone and eplerenone were cost-effective, with incremental cost-effectiveness ratios (ICER) in international dollars (Int$) of 7,955/QALY and Int$ 6,459/QALY, respectively. Dapagliflozin and sacubitril-valsartan showed ICERs of Int$ 9,000/QALY and Int$ 11,691/QALY, respectively, and were also considered cost-effective. Cardiac resynchronization therapy was classified as highly cost-effective (ICER Int$ 15,723/QALY), whereas the implantable cardioverter-defibrillator for primary prevention did not demonstrate cost-effectiveness (ICER Int$ 50,345/QALY). Several HF interventions show economic attractiveness. However, most technologies were evaluated in only one study, which limits more robust analyses. Expanding national economic studies is necessary to support access to effective therapies while maintaining the sustainability of the health system.

## Introduction

Heart failure (HF) is one of the leading causes of hospitalization and cardiovascular mortality in Brazil. In 2021, direct costs attributable to HF exceeded R$ 22 billion in the Brazilian Unified Health System (SUS), reflecting not only the high prevalence of the condition but also the increasing complexity of its clinical management. Between 2008 and 2021, more than 3.4 million hospitalizations for HF were recorded in the SUS, corresponding to more than one third of clinical admissions due to cardiovascular causes. In parallel, an increase was observed in high-complexity procedures, such as revascularizations, valve replacements, arrhythmia ablations, and device implantations, highlighting the progression of case severity and the rise in costs associated with technologies used in HF management. Indirect costs, including productivity losses and social security benefits, are also substantial, estimated at approximately R$ 6 billion per year.^1 , 2^

Despite the availability of new therapies, access to and adherence to treatment remain limited, largely due to the cost of chronic and continuous medications as well as procedure costs.^2^According to Brazilian health system regulations, recommendations for incorporating therapeutic technologies must consider not only efficacy, safety, and effectiveness but also cost-effectiveness to support informed and efficient decision-making. Although economic studies exist, there is still no structured synthesis consolidating evidence on the cost-effectiveness of therapies applied to HF within the context of the Brazilian health system.

Because of the magnitude of the problem and the need for rational resource allocation, this study was conducted to support the recommendation process of the Brazilian Guideline for Chronic and Acute Heart Failure, developed by the Brazilian Society of Cardiology. Thus, a systematic review of economic analyses on HF therapies in Brazil was performed.

## Methods

### Search strategy

A high-sensitivity search was conducted in the PubMed, Embase, LILACS, and SciELO databases to identify Brazilian studies that evaluated costs and cost-effectiveness of pharmacological and nonpharmacological therapies in HF management, published up to 18 March 2025. Nonpharmacological interventions included procedures and devices.

Medical Subject Headings (or equivalent terms in the other databases) were used, such as “economics”, “costs and cost analysis”, “value of life”, and “heart failure”, in addition to free terms including “cost”, “price”, “pharmacoeconomic”, “budget”, and “money value”.

### Eligibility criteria

Studies were considered eligible when they met the following criteria: i) population composed of individuals diagnosed with HF, regardless of ejection fraction (EF), age, sex, or etiology; ii) intervention corresponding to any validated therapy for HF management; iii) comparison with another active treatment, placebo, or absence of intervention; iv) outcomes related to disease cost in international dollars (Int$), cost per quality-adjusted life year (QALY), and cost per clinical outcomes (e.g., hospitalization and mortality); v) design including economic analyses of cost-effectiveness, cost-utility, or cost-of-illness.

Reviews, case reports, letters to the editor, editorials, studies without explicit economic analysis, studies without relevant data for this review, and studies conducted outside the Brazilian setting were excluded from the analysis.

### Study selection and data extraction

Study selection was performed using the Covidence platform.^3^After duplicate removal, four independent review authors screened titles and abstracts. Eligible articles underwent full-text review by two independent review authors. Disagreements regarding inclusion or exclusion were resolved by consensus or by consultation with a third review author.

### Assessment of methodological quality

The methodological quality of the included studies was assessed using the Consensus on Health Economic Criteria (CHEC) list,^4^composed of 19 items covering essential aspects of trial-based economic evaluations. Each item was classified as “Yes”, “No”, or “Not applicable”, with a justification recorded. The assessment was conducted independently by two review authors, with disagreements resolved by consensus or by a third review author.

### Presentation of findings

Because of the methodological heterogeneity observed among the included studies, results were synthesized narratively, including description of the analytical perspective, methods used, data sources, and main findings. Studies were grouped into pharmacological and nonpharmacological therapies, and the quality of evidence for these interventions was described in detail.

Analysis of incremental cost-effectiveness ratio (ICER) results in relation to the willingness-to-pay (WTP) threshold was conducted qualitatively. Costs were expressed in Int$, adjusted by purchasing power parity (PPP) corresponding to the year each study was conducted, without application of inflation correction. Values were interpreted relative to gross domestic product (GDP) per capita and the WTP threshold in effect at the year of publication.

In 2024, Brazil’s GDP per capita adjusted by PPP was 19,648 Int$. Official sources included the Brazilian Institute of Geography and Statistics for per capita values in Brazilian reais over the last 25 years^5^and trade marketing data for conversion to Int$.^6^

Regarding the WTP threshold, in accordance with the guidance document of the National Committee for Health Technology Incorporation in the Brazilian Unified Health System (CONITEC, in Portuguese),^7^therapies with values below 1 times GDP per capita were considered cost-effective. Values between 1 and 3 times GDP per capita were classified as cost-effective in special scenarios, such as absence of therapeutic alternatives, severe diseases, rare diseases, or oncological conditions. Values above 3 times GDP per capita were considered indicative of lack of cost-effectiveness.

## Results

### Characteristics of selected studies

Searches identified 784 articles. After duplicate removal and title and abstract screening, 66 publications were selected for full-text review. Of these, 25 were included in the systematic review ( Figure 1 ). Among the included studies, 10 evaluated cost of illness and 13 investigated cost-effectiveness ( Central Illustration ), including three CONITEC documents as well as one literature review and one systematic review.

**Figure 1:** – Flowchart of study selection for review.

### Pharmacological treatment of heart failure

Several studies were identified that evaluated the cost-effectiveness of therapies considered essential in HF management. Table 1 describes the characteristics of economic analyses of pharmacological therapies conducted in Brazil from the SUS perspective. Regarding medications that reduce mortality in HF with reduced EF (HFrEF), studies involving mineralocorticoid receptor antagonists (MRAs), sodium-glucose cotransporter 2 inhibitors (SGLT2is), and angiotensin receptor-neprilysin inhibitors (ARNIs) were selected. No studies were identified in the context of HF with preserved EF (HFpEF).

**Table 1:** – Economic analyses of pharmacological therapy in patients with HF in Brazil

Study ID	Economic analysis	Population	Intervention	Comparison	Outcomes	Perspective
CONITEC, 2018^11^	Cost-effectiveness	≥ 18 years, HF NYHA II-IV and LVEF ≤ 35%	Sacubitril/valsartan	Standard treatment with ACEis or ARBs	ICER of Int$ 11,691/QALY	Public health care system
CONITEC, 2022^7^	Cost-effectiveness	≥ 18 years, HF NYHA II-IV with LVEF ≤ 40%, on standard treatment with ACEis/ARBs, beta-blockers, diuretics, and MRAs	Dapagliflozin	Standard treatment (ACEis/ARBs, beta-blockers, diuretics, and MRAs)	ICER of Int$ 3,907/QALY	Public health care system
Koeche et al., 2025^8^	Cost-effectiveness	≥ 18 years with HFrEF, NYHA II-IV	Spironolactone	Standard treatment	ICER of Int$ 7,955/QALY	Public health care system
			Eplerenone		ICER of Int$ 6,459/QALY	
			Finerenone		ICER of Int$ 109,840/QALY	
Naves et al., 2025^9^	Cost-effectiveness	≥≥ 18 years, HF NYHA II-IV with LVEF ≤ 40%, on standard treatment with ACEis/ARBs, beta-blockers, diuretics, and MRAs	Dapagliflozin	Standard treatment	ICER of Int$ 9,000/QALY	Public health care system

ACEis: angiotensin-converting enzyme inhibitors; ARBs: angiotensin ii receptor blockers; HF: heart failure; HFrEF: HF with reduced ejection fraction; ICER: incremental cost-effectiveness ratio; Int$: international dollar; LVEF: left ventricular ejection fraction; MRAs: mineralocorticoid receptor antagonists; NYHA: New York Heart Association; QALY: quality-adjusted life year.

Spironolactone, eplerenone, and finerenone were evaluated in patients with HFrEF of ischemic and nonischemic etiology in one cost-effectiveness study.^8^Among MRAs, spironolactone and eplerenone were considered cost-effective strategies, with cost per QALY gained — or ICER — of Int$ 7,955/QALY and Int$ 6,459/QALY, respectively, values below the WTP corresponding to 1 times GDP per capita. In probabilistic sensitivity analyses, spironolactone and eplerenone demonstrated robustness, with probability of cost-effectiveness in 87% and 92% of iterations, respectively. In contrast, finerenone was considered not cost-effective, with an ICER of Int$ 109,840/QALY, substantially above the WTP adopted by the Ministry of Health.

Among SGLT2is, dapagliflozin was economically favorable regardless of the presence of diabetes mellitus. Incorporation into standard HFrEF treatment resulted in an additional gain of 0.36 QALY and an ICER of Int$ 9,000/QALY. The acceptability curve analysis indicated that, considering a WTP of Int$ 17,250, the probability of cost-effectiveness was 96.6%.^9^

Economic evaluation of ARNIs was based on the Latin American subpopulation of the randomized clinical trial (RCT) PARADIGM-HF,^10^aiming for greater representativeness for the Brazilian population. Compared with enalapril, ARNI use provided a gain of 0.57 QALY and an ICER of Int$ 11,691/QALY, being classified as a cost-effective strategy.^11^Value-based recommendations for pharmacological therapies are presented in Table 2 .

**Table 2:** – Cost-effectiveness and health value in pharmacological therapy for HFrEF

Therapy	Qualitative cost-effectiveness	Level of certainty	Recommendation
Spironolactone and eplerenone	Dominant or ICER < Int$ 19,648*/QALY	High	High health value in the treatment of patients with symptomatic HFrEF
Finerenone	ICER > Int$ 58,944^†^/QALY	Low	Low health value in the treatment of patients with symptomatic HFrEF
Dapagliflozin	Dominant or ICER < Int$ 19,648*/QALY	High	High health value in the treatment of patients with symptomatic HFrEF
Sacubitril + valsartan	Dominant or ICER < Int$ 19,648*/QALY	High	High health value in the treatment of patients with symptomatic HFrEF

*GDP per capita in Brazil in 2024 adjusted by PPP.^†^Three times GDP per capita in Brazil in 2024 adjusted by PPP. GDP: gross domestic product; HFrEF: heart failure with reduced ejection fraction; ICER: incremental cost-effectiveness ratio; Int$: international dollar; PPP: purchasing power parity; QALY: quality-adjusted life year.

To date, no national economic analyses have been identified for other mortality-reducing pharmacological therapies in HFrEF, such as angiotensin-converting enzyme inhibitors (ACEis), beta-blockers, and angiotensin II receptor blockers (ARBs).

In addition to pharmacological costs, HF patient follow-up involves diagnostic tests, health professional fees, transportation, and health care infrastructure. A national study published in 2024, which used time-driven activity-based costing, demonstrated mean costs of Int$ 398 for patients in New York Heart Association (NYHA) functional class I, Int$ 548 for NYHA II, and Int$ 1,233 for NYHA III/IV, with laboratory and imaging tests representing up to 74% of expenditures in class III/IV.^12^

### Nonpharmacological treatment of heart failure

Regarding nonpharmacological interventions ( Table 3 ), cardiac rehabilitation, or supervised exercise, is safe in patients with stable HF. In the Kühr et al.,^13^exercise three times per week for 3 months was assumed, followed by one weekly session for 9 months, totaling 72 sessions in the 1st year. Compared with usual care, cardiac rehabilitation showed an ICER of Int$ 26,462/QALY. In Monte Carlo sensitivity analysis, considering a WTP of Int$ 27,500, the intervention was cost-effective in 55% of iterations.

**Table 3:** – Economic analyses of nonpharmacological therapy in patients with HF in Brazil

Study ID	Economic analysis	Population	Intervention	Comparison	Outcomes	Perspective
Kühr et al., 2011^13^	Cost-effectiveness	≥ 60 years, HF NYHA II-III	Supervised exercise	Standard treatment	ICER of Int$ 26,462/QALY	Public health care system
Ruschel et al., 2018^15^	Cost-effectiveness	≥ 18 years, EF ≤ 45%, hospitalization for decompensated HF	Home visits and telephone calls by nurses	Standard treatment	ICER of Int$ 265 per readmission avoided	Public health care system
Ruschel et al., 2018^15^	Cost-effectiveness	≥ 18 years, EF ≤ 45%, hospitalization for decompensated HF	Home visits and telephone calls by nurses	Standard treatment	Dominant (lower cost and more effective)	Private health care system
Ghisleni et al., 2023^12^	Cost of illness	≥ 18 years, HF with EF < 50%	Outpatient visits and diagnostic tests	—	Mean cost in 6 months: Int$ 398 (NYHA I); Int$ 548 (NYHA II); Int$ 1,233 (NYHA III/IV)	Public health care system
Goldraich et al., 2018^18^	Cost of illness	Advanced HF, heart transplant recipients	Heart transplantation	—	Mean cost of Int$ 107,390 per transplanted patient	Public health care system
Ribeiro et al., 2010^20^	Cost-effectiveness	≥ 60 years, EF < 35%, HF NYHA II-III	ICD (primary prevention)	Standard treatment	ICER of Int$ 50,345/QALY	Public health care system
Bertoldi et al., 2013^21^	Cost-effectiveness	EF < 35%, HF NYHA II-IV, bundle branch block	CRT	Standard treatment	ICER of Int$ 15,723/QALY	Public health care system
Bertoldi et al., 2013^21^	Cost-effectiveness	EF < 35%, HF NYHA II-IV, bundle branch block	ICD and upgrade to CRT	ICD	ICER of Int$ 36,940/QALY	Public health care system
Wherry et al., 2021^22^	Cost-effectiveness	Patients at higher arrhythmic risk*	ICD	Standard treatment	ICER of Int$ 9,575/QALY	Public health care system
Decker et al., 2024^17^	Cost-effectiveness	≥ 18 years, refractory cardiogenic shock	VA-ECMO	Standard treatment	ICER of Int$ 37,491/QALY; ICER of Int$ 27,432 per life year gained	Public health care system

*Patients with at least one of the following variables: nonsustained ventricular tachycardia, frequent ventricular ectopy, EF < 25%, or presyncope/syncope. CRT: cardiac resynchronization therapy; EF: ejection fraction; HF: heart failure; ICD: implantable cardioverter-defibrillator; ICER: incremental cost-effectiveness ratio; Int$: international dollar; NYHA: New York Heart Association; QALY: quality-adjusted life year; VA-ECMO: venoarterial extracorporeal membrane oxygenation.

The strategy of regular home visits performed by trained nurses, combined with telephone contact for 24 weeks, was evaluated in an RCT in Brazil and demonstrated a 27% reduction in the composite outcome of death, emergency visits, or readmission.^14^From the SUS perspective, the intervention showed an ICER of Int$ 265 per readmission avoided; from the private health care perspective, it was dominant, with lower cost and lower readmission rate.^15^

In critically ill patients with imminent risk of death, WTP up to 3 times GDP per capita may be accepted. In refractory cardiogenic shock, escalation to mechanical circulatory support devices may occur, including venoarterial extracorporeal membrane oxygenation (VA-ECMO). A recent RCT did not demonstrate mortality benefit with VA-ECMO in post-myocardial infarction cardiogenic shock compared with standard therapy,^16^maintaining debate regarding its indication. In an economic analysis conducted in Brazil from the SUS perspective, VA-ECMO use in patients with cardiogenic shock showed an ICER of Int$ 37,491/QALY compared with standard therapy, considering WTP values between 1-3 times GDP per capita. This result was particularly sensitive to hospitalization cost and survival probability of patients receiving VA-ECMO.^17^

In patients with advanced HF, a cost study conducted in 2018 with 27 heart transplant recipients in a reference center identified a mean cost of Int$ 107,390 per patient, considering hospitalization associated with transplantation. This value exceeded SUS reimbursement per patient by approximately 60%.^18^

The use of cardiac devices has increased in HF management. Studies on implantable cardioverter-defibrillator (ICD), conducted from the SUS and private health care perspectives, indicated low economic attractiveness in primary prevention of ventricular arrhythmias, regardless of etiology. However, ICD was cost-effective when indicated for individuals at higher arrhythmic risk, especially in ischemic HF and in secondary prevention.^19 , 20^

Regarding cardiac resynchronization therapy (CRT), a study from the SUS perspective demonstrated that CRT alone is highly cost-effective in patients with HF, EF < 35%, NYHA functional class II-IV, and dyssynchrony (left bundle branch block). Upgrade to CRT combined with ICD was not cost-effective in the overall population, requiring careful selection of patients at higher arrhythmic risk.^21^

An additional economic analysis evaluated ICD implantation in a subgroup of patients with additional risk factors, including nonsustained ventricular tachycardia, frequent ventricular extrasystoles, EF < 25%, or syncope/presyncope. In this higher-risk group, ICD was cost-effective, with cost/QALY below usual WTP thresholds.^22^

### Quality of evidence

Methodological assessment of the 25 studies revealed variability in quality. 6 studies met 18 of the 19 CHEC checklist criteria, corresponding to 94.7% conformity, while 3 reached 89.4%, indicating good adherence to principles of economic evaluation. Lower methodological quality observed in part of the studies is related to the fact that 12 were not full cost-effectiveness analyses, which limits applicability of several CHEC list items. Additional details on methodological quality assessment are presented in the Supplementary Appendix.

## Discussion

The incorporation of new therapies into the SUS depends on economic analyses conducted according to criteria established by CONITEC, constituting a mandatory component of technology assessment dossiers. The SUS perspective generally adopts a medium- to long-term time horizon, considering direct system costs and impacts on clinical outcomes, such as hospitalizations, mortality, and quality of life, measured by QALY or disability-adjusted life years. Recently, CONITEC has adopted cost-effectiveness thresholds close to 1 times GDP per capita (R$ 40,000/QALY in 2022) as high (desirable) health value and close to 3 times GDP per capita (R$ 120,000/QALY in 2022) as low value, with intermediate values between these references.^7^Both regulatory bodies have progressively incorporated value-based health care principles to promote greater efficiency, equity, and rational resource allocation.

Pharmacological treatment of HF aims primarily to reduce mortality, hospitalizations, and symptoms. In the context of HFrEF, therapies with consistent impact on these outcomes include ACEis, ARBs, ARNIs, beta-blockers, MRAs, and SGLT2is. Due to the substantial clinical benefit of these interventions, most cost-effectiveness analyses focus on HFrEF. In Brazil, studies involving MRAs, SGLT2i, and ARNI demonstrated ICER below 1 times GDP per capita, indicating that these therapies provide high health value in the national setting.

Conversely, in HFpEF, the historical scarcity of therapies with consistent impact on relevant clinical outcomes contributes to the absence of specific economic evaluations in the country. Recent evidence involving SGLT2i, finerenone, and weight-reduction therapies suggests that economic studies in this population are likely to emerge in the coming years.

Despite well-established efficacy of beta-blockers, ACEi, and ARB in reducing cardiovascular outcomes, no economic analyses of these therapies were identified in the Brazilian context. In other countries, evaluations conducted in the 1990s and 2000s showed favorable results. Enalapril, captopril, and ramipril were economically viable options in HFrEF; specifically, enalapril showed an ICER of US$ 115/QALY compared with placebo in patients with left ventricular ejection fraction (LVEF) ≤ 35%.^23^Similarly, valsartan use in post-infarction systolic dysfunction demonstrated an ICER of £ 5,300/QALY, below the WTP adopted in the United Kingdom.^24^

Economic evaluations with beta-blockers were conducted in the United States, Italy, and the United Kingdom. In one analysis, metoprolol succinate showed an ICER of US$ 4,200/QALY compared with placebo,^25^demonstrating cost-effectiveness, including in individuals aged > 70 years.^26^

Other adjunct therapies in symptomatic HFrEF have also been evaluated. In the US health care system, the combination of hydralazine and isosorbide dinitrate in Black patients with symptomatic HFrEF was a dominant strategy, meaning more effective and less costly than conventional treatment.^27^In the United Kingdom, IV ferric carboxymaltose replacement in patients with symptomatic HFrEF and iron deficiency, with or without anemia, showed an ICER of € 4,414/QALY, substantially below the WTP adopted in the country (€ 22,200-33,300/QALY).^28^

Ivabradine, indicated for patients with HFrEF NYHA functional class II-III in sinus rhythm, heart rate ≥ 70 bpm, and use of maximally tolerated beta-blocker dose,^29^was evaluated in a US study with an estimated ICER of US$ 28,318/QALY and was classified as an intermediate-value intervention.^30^Vericiguat, a soluble guanylate cyclase stimulator, demonstrated reduction in the composite outcome of cardiovascular death and HF hospitalization in the VICTORIA study.^31^A subsequent economic analysis estimated an ICER of US$ 124,512/QALY, classifying it as an intermediate-value therapy in the US population.^32^

Regarding digoxin, a study from the 1990s in the United States indicated cost reduction with its maintenance compared with standard therapy at that time. However, interpretation of these results should be cautious, since therapies currently considered pillars of HFrEF treatment, such as beta-blockers and MRAs, were not yet widely used in that period.^33^

In Brazil, costs related to heart transplantation are particularly relevant. The mean cost of Int$ 107,390 per patient is similar to that observed in Europe and approximately half the mean value reported in the United States. At the time of publication, Ministry of Health reimbursement was Int$ 26,010 per procedure, substantially lower than the actual cost.^18^

A multidisciplinary approach is essential for engagement and adherence of patients with HF. Strategies such as post-discharge home visits by trained professionals and cardiac rehabilitation programs have been widely recommended. Although an RCT demonstrated modest reduction in mortality and hospitalization only after adjustment for prognostic variables,^34^a Brazilian economic analysis indicated cost per QALY between 1-3 times GDP per capita, which represents intermediate health value.

Economic evaluation of implantable devices presents specific challenges. These devices may provide relevant clinical benefit across a broad range of patients; however, their cost-effectiveness varies according to clinical profile and baseline risk of the population. In addition, the non-linear cost pattern, with high initial expenses related to implantation, complicates indiscriminate adoption in health care systems.

Economic studies on ICD and CRT in Brazil were mostly published in the 2010s. Technological advances and changes in standard treatment since then may modify the cost-effectiveness of these interventions, both due to impact on costs and device durability and due to changes in patient baseline risk.

To the best of our knowledge, this is the first systematic review of economic analyses on HF therapies in Brazil. There is growing recognition of the role of cost-effectiveness analyses as a complement to clinical efficacy data in the incorporation of interventions in public and private systems. Nevertheless, important gaps remain in national economic evidence production in HF.

### Study limitations

This study has some limitations. First, for most therapies there is only 1 available economic analysis, which reduces robustness of conclusions and limits generalization to different population profiles and care settings. This scarcity also restricts application of evidence certainty assessment methodologies (e.g., Grading of Recommendations Assessment, Development and Evaluation) that depend on multiple sources.

Second, several models used international data to estimate efficacy outcomes, especially in older analyses. Necessary adaptations in this process require caution when extrapolating results, considering differences among health care systems, epidemiology, and treatment adherence.

Finally, absence of cost-effectiveness analyses in HFpEF in Brazil prevents specific recommendations for this subgroup.

## Conclusion

This systematic review demonstrates that multiple interventions with clinical impact in HF show economic attractiveness, with favorable cost per QALY ratios even in budget-constrained scenarios. However, an important gap was identified in the availability of economic analyses conducted from the Brazilian perspective. Expanding production of national economic studies may support equitable access to the best available interventions, promote rational resource use, and contribute to sustainability of health care systems while improving patient clinical outcomes.
